# Ononitol Monohydrate—A Glycoside Potentially Inhibit HT-115 Human Colorectal Cancer Cell Proliferation through COX-2/PGE-2 Inflammatory Axis Regulations

**DOI:** 10.3390/ijms232214440

**Published:** 2022-11-21

**Authors:** Pandurangan Subash-Babu, Alanoud Aladel, Taghreed N. Almanaa, Sahar Abdulaziz AlSedairy, Ali A. Alshatwi

**Affiliations:** 1 Cancer Molecular Biology Research Lab, Department of Food Science and Nutrition, College of Food and Agricultural Sciences, King Saud University, Riyadh 11451, Saudi Arabia; 2 Community Health Sciences, College of Applied Medical Sciences, King Saud University, Riyadh 11451, Saudi Arabia; 3 Department of Botany and Microbiology, College of Science, King Saud University, Riyadh 11451, Saudi Arabia

**Keywords:** ononitol monohydrate, inflammation, COX-2/PGE-2, colorectal cancer, apoptosis

## Abstract

We aimed to inhibit HT-115 human colorectal cancer cell proliferation using ononitol monohydrate (OMH), a bioactive principle isolated from *Cassia tora* (L.). The cytotoxicity of OMH has been assayed using MTT (3-(4,5-dimethylthiazol-2-yl)-2,5-diphenyl tetrazolium bromide), cell and nuclear morphology, and apoptosis mechanisms have been analyzed using real-time PCR. Higher doses of OMH potentially inhibit 84% of HT-115 cell viability; we observed that the IC_50_ level was 3.2 µM in 24 h and 1.5 µM in 48 h. The treatment with 3.2 µM of OMH for 48 h characteristically showed 64% apoptotic cells and 3% necrotic cells, confirmed by propidium iodide and acridine orange/ethidium bromide (AO/ErBr) staining. We found the overexpression of cyclooxygenase-2 (COX-2) and prostaglandin E2 (PGE-2) in the control HT-115 cells, which was directly associated with colorectal tumorigenesis. However, 3.2 µM of OMH treatment to HT-115 cells for 48 h significantly reduced inflammatory genes, such as TNF-α/IL-1β and COX-2/PGE-2. The downregulation of COX-2 and PGE-2 was more significant with the 3.2 µM dose when compared to the 1.5 µM dose of OMH. Additionally, the protein levels of COX-2 and PGE-2 were decreased in the 3.2 µM OMH-treated cells compared to the control. We found significantly (*p* ≤ 0.01) increased mRNA expression levels of tumor-suppressor genes, such as pRb2, Cdkn1a, p53, and caspase-3, and decreased Bcl-2, mdm2, and PCNA after 48 h was confirmed with apoptotic stimulation. In conclusion, the antiproliferative effect of OMH via the early suppression of protumorigenic inflammatory agents TNF-α/IL-1β, COX-2/PGE-2 expression, and the increased expression levels of tumor-suppressor genes Cdkn1a and pRb2, which enhanced the activation of Bax and p53.

## 1. Introduction

Chronic unhealthy dietary patterns initiate abnormal mucosal immune responses in the intestine, leading to inflammatory bowel disease (IBD) [1]. The early progression of colorectal cancer (CRC) is associated with multiple factors, such as poor nutrition, dietary pattern, chronic intestinal inflammation, and IBD [2]. In addition, the dietary pattern alters the intestinal microbiota associated with proinflammatory signaling and cytokine-transcription factor synthesis, which initiate colorectal tumorigenesis [3]. Chronic intestinal inflammation secretes cytokines and chemokines, which regulate malignancies by stimulating angiogenesis and inhibiting immune-mediated tumor suppression [4].

In colorectal tumorigenesis, cell proliferation is fundamental, and this process is regulated by an important enzyme, cyclooxygenases (COXs) [5]. In humans, COX-1 is responsible for cellular homeostasis, and COX-3 is responsible for thermal regulation. In addition, COX-2 regulates dysplastic and metaplastic tissue growth, as well as the expansion and progression of colon cancer [6,7]. Chronic gut inflammation alters the innate/adaptive immune system and the integrity of intestinal epithelia cell proliferation and function. Proinflammatory and tumorigenesis factors such as IL-1β, TNF-α, and COX-2 activate Nf-κB, a key inhibitor of malignant cell apoptosis [8]. In addition, COX-2 inhibits apoptosis via the mitogen-activated protein kinase or phosphoinositide 3-kinase-AKT signaling pathways, which increase the expression of Bcl-2, further initiating uncontrolled colonic tumorigenesis [9].

COX-2-regulated prostaglandin synthesis was considered to be an important factor in colorectal tumorigenesis [10]. The catalytic action of cyclooxygenases converts free arachidonic acid to prostaglandin H2; it is the precursor of other prostaglandins and thromboxane. Prostaglandins and thromboxane are the regulatory proteins that control numerous biological processes, such as cell growth, development, and pro- and anti-inflammatory function, which are all vital to the growth and angiogenesis of neoplasms [6]. Enhanced COX-2 expression results in excessive levels of its principal metabolic product, PGE-2; this pleiotropic effect has been identified under the influence of different types of cancer growth [11]. 

PGE-2 also activates β-catenin-dependent signaling, which promotes the survival and proliferation of cancer cells [12]. COX-2 expression is upregulated in colorectal tumors and in the experimental models of CRC [13]. The protumorigenic effects of COX-2 are mediated by its major end product, PGE2, and increased levels of PGE-2 in human CRC have been confirmed by many studies [14,15]. Alterations in the COX-2/PGE-2 inflammatory axis are considered to be an important component of colorectal malignancies or sporadic CRC [16]. At present, the treatment available for IBD or CRC is critical because COX-2 inhibitors might aggravate colitis and colonic injury. 

We aimed to identify a natural agent that is non-toxic and cancer-cell-specific cytotoxic with a mitochondria-dependent apoptotic mechanism. Glycoside ononitol monohydrate (OMH) was isolated from *Cassia tora* (L), as confirmed in our earlier study [17]. Previously, steviol glycosides and cardiac glycosides, antioxidants from natural origins, have been identified as potential therapeutic agents against gastrointestinal cancer and a variety of chronic diseases [17,18,19]. Cerberin (CR), a cardenolide isolated from the fruit kernel of *Cerbera odollam*, has been identified to effectively inhibit cancer cell growth [20]. In our previous study, we observed that OMH is biologically safe, as confirmed by no toxicity in human mesenchymal stem cells, and potentially inhibits inflammatory cytokine development in macrophages and regulates lipid metabolism in adipocytes [21]. In the present study, we aimed to suppress pro-tumorigenic inflammatory cytokine COX-2/PGE-2 expression using ononitol monohydrate (OMH), further controlling the regulation of cytokine transcription factor and proinflammatory signaling networks, which inhibits CRC progression.

## 2. Results

### 2.1. In Vitro Cytotoxic Effect of OMH

**Figure 1:**
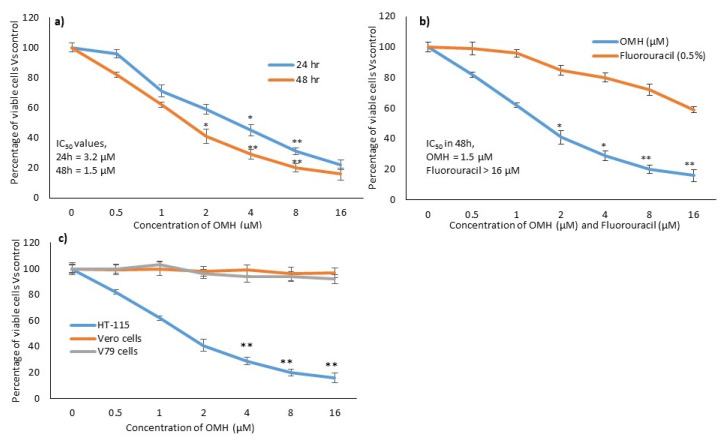
In vitro cytotoxic potential of ononitol monohydrate (OMH) on HT-115 cells after 24 h and 48 h (**a**). (**b**), showing the relative cytotoxic effect of OMH and fluorouracil in HT-115 cells after 48 h. (**c**), showing the comparative cytotoxic effect of OMH on HT-115, Vero, and V79 cells after 48 h. Data are expressed as the mean ± SD (*n* = 6). * *p* ≤ 0.01 vs. Control, ** *p* ≤ 0.05 vs. control. For (**c**), ** *p* ≤ 0.05 vs. Vero and V79 cells.

The cytotoxicity assay indicated that the HT-115 cells treated with OMH resulted in significant inhibition of cell growth and cytotoxicity (Figure 1). The cell viability significantly (*p* ≤ 0.05) decreased to 84% in the tested higher concentration of OMH (16 µM). The IC_50_ level of the OMH-treated HT-115 cells was found to be at 1.5 µM in 48 h and 3.2 µM in 24 h (Figure 1a,b). The reference drug, fluorouracil, at a 3.2 µM concentration, only inhibited 17% of HT-115 cell viability after 48 h. Therefore, 1.5 µM and 3.2 µM of OMH were selected as the testing concentration for the subsequent in vitro morphological apoptosis and molecular-level gene expression analysis. Figure 1c shows the selective cytotoxicity of OMH against HT-115 colon cancer cells when compared to the Vero and V79 cells. Most notably, the tested higher concentration of OMH (16 µM) inhibited 8% and 11% of cell growth in the Vero and V79 cells, respectively. 

### 2.2. Effect of OMH on Cell and Nuclear Morphology

**Figure 2:**
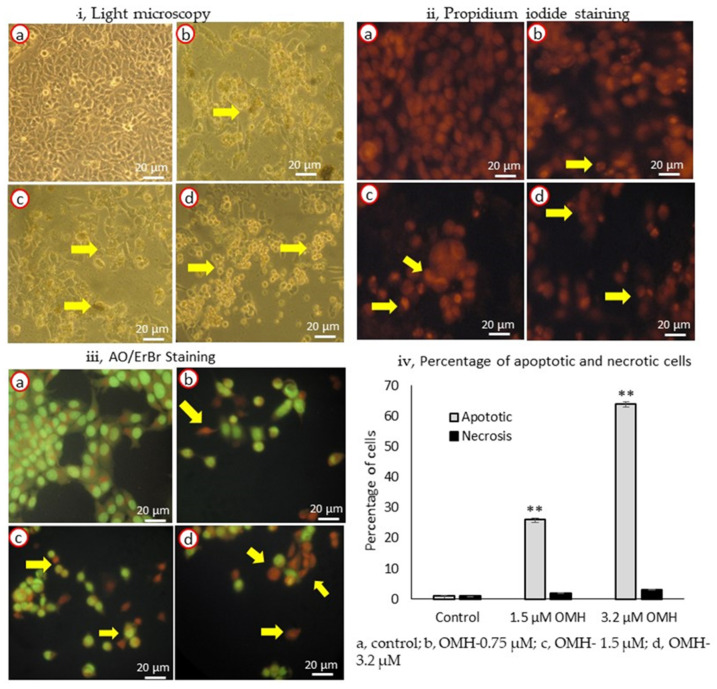
Light microscopy (**i**), propidium iodide (**ii**), AO/ErBr-stained (**iii**) images (40×) [(**a**), untreated HT-115 cells; (**b**), 0.75 µM of OMH treatment; (**c**), 1.5 µM of OMH treatment; and d, 3.2 µM of OMH-treated HT-115 cells] and the percentage of apoptotic and necrotic cells in 1.5 and 3.2 µM of OMH-treated HT-115 cell (**iv**) after 48 h. (**ii**) PI staining of 1.5 µM (**c**) and 3.2 µM (**d**) of OMH-treated HT-115 cells, showing stressed cells with nuclear linearization and cell death was evidenced by the morphology of abnormal nuclei and horseshoe-shaped nucleus. However, 0.75 µM dose of OMH was not significant to apoptosis stimulation. (**iii**) AO/ErBr staining of 3.2 µM of OMH-treated HT-115 cells (**d**) showing proapoptotic (bright green), early apoptotic (light green), late apoptotic (orange), and necrotic cells (red) are shown by arrowheads. The observed effect was higher in 3.2 µM when compared to 1.5 µM of OMH. In 0.75 µM dose of OMH the majority of cells were intact and normal. (**iv**) Data presented as the means ± SD are shown; *n* = 6; values sharing a common superscript as ** *p* ≤ 0.01 compared with control.

The apoptosis- and necrosis-associated morphological variations in the OMH-treated HT-115 cells are shown in Figure 2. Figure 2i shows the light microscopy analysis of the control, 0.75, 1.5, and 3.2 µM doses of the OMH-treated HT-115 cells after 48 h. We clearly observed the normal and uniform morphology of the control cells, but the OMH-treated cells showed abnormal and varying sizes of HT-115 cells. In PI staining, we observed the characteristic irregular and horseshoe-shaped nuclei, which confirmed the nuclear damage and initiation of apoptosis (Figure 2ii,b–d). 

In AO/ErBr staining, the 1.5 and 3.2 µM doses of OMH showed the presence of pre-apoptotic cells (dark green), early apoptotic (light green), late apoptotic (orange), and necrotic cells (red) (Figure 2iii, d) when compared to the control (Figure 2iii,a) or the 1.5 µM dose (Figure 2iii,c); however, the 3.2 µM dose of OMH showed higher numbers of late-apoptotic HT-115 cells (Figure 2iii,d). The HT-115 cells treated with 3.2 µM of OMH indicated that 64% of the cells were apoptotic and 3% were necrotic cells. The increase in apoptotic cells was dose-dependent, being significantly (*p* ≤ 0.01) higher (64%) in 3.2 µM when compared to 1.5 µM (12%) and 0.75 µM (3%) of OMH treatment. In 3.2 µM of OMH, it was shown there were 3% necrotic cells after 48 h (Figure 2iv). The lower dose, 0.75 µM, was not found to be significant to the stimulation of apoptosis. So, the 3.2 µM and 1.5 µM doses of OMH were selected as effective doses and used for further analysis.

### 2.3. Effect of OMH on Inflamamtory and Protumorigenic Gene Expression Levels

**Figure 3:**
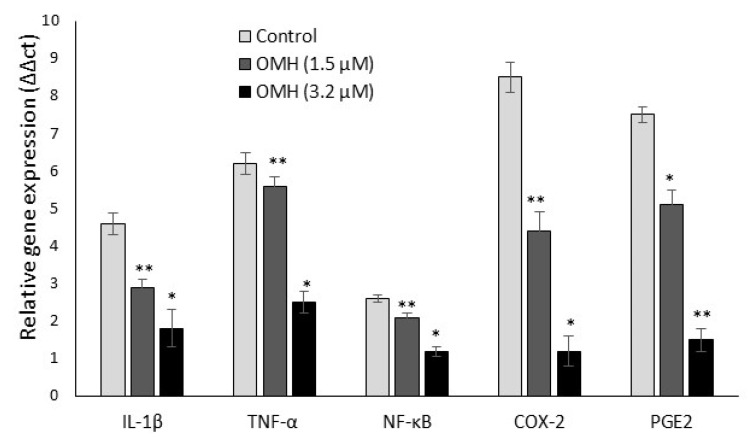
Effect of OMH on proinflammatory and protumorigenic gene expression levels in HT-115 cells after 48 h. Data presented as the means ± SD (*n* = 6). Values sharing a common superscript as * *p* ≤ 0.01 and ** *p* ≤ 0.05 compared with untreated HT-115 cells.

Figure 3 shows the effect of OMH on inflammatory (IL-1β, TNF-α, and NF-κB) and protumorigenic (COX-2 and PGE-2) mRNA expression levels in the HT-115 cells after 48 h. The decreased gene expression levels of protumorigenic COX-2 were two-fold; PGE-2 and TNF-α were one-fold in the 3.2 µM OMH treatment when compared to 1.5 µM. The OMH treatment decreased the expression levels of COX-2, PGE-2, and TNF-α significantly (*p* ≤ 0.01) when compared to the control cells. In addition, we observed that the mRNA expression levels of IL-1β and NF-κB also significantly decreased with the 3.2 µM dose of OMH-treated cells when compared to the 1.5 µM of OMH and the control.

### 2.4. Effect of OMH on Oxidative Stress and Apoptotic Gene Expression

**Figure 4:**
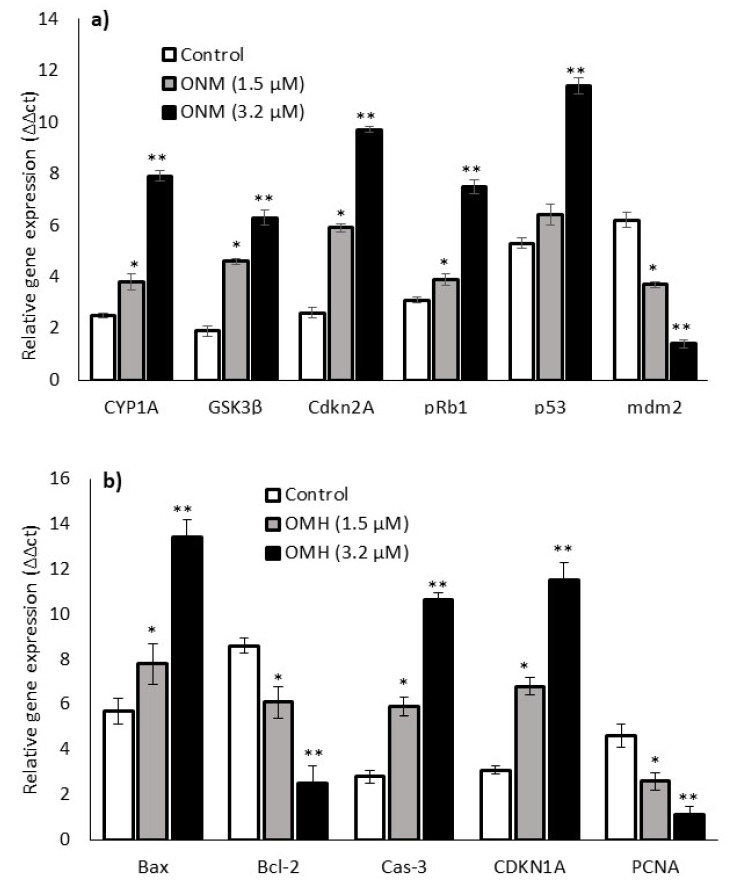
Effect of OMH on antioxidant, tumor suppressor (**a**), and apoptosis-related (**b**) gene expression levels of OMH-treated HT-115 cells after 48 h. Data presented as the means ± SD (*n* = 6). Values sharing a common superscript as * *p* ≤ 0.01 and ** *p* ≤ 0.05 compared with untreated HT-115 cells.

Figure 4 shows the oxido-reductase (CYP1A and GSK3β), tumor suppressor (Cdkn2A, pRb1, p53, and mdm2), and apoptosis (Bax, Bcl-2, Caspase 3, CDKN1A, and PCNA)-related mRNA expression levels after 48 h in 1.5 µM and 3.2 µM of OMH-treated HT-115 cells. The expression levels of CYP1A, GSK3β, Cdkn2A, pRb1, p53, Bax, Caspase 3, and CDKN1A increased one-fold, and mdm2, Bcl-2, and PCNA were decreased after 48 h in the OMH-treated HT-115 cells. Most notably, the mRNA expression levels of oxidative stress (CYP1A and Cdkn2A) and tumor suppressor (pRb1 and p53) genes were significantly (*p* ≤ 0.05) higher in 3.2 µM of OMH when compared to 1.5 µM of OMH treatment after 48 h (Figure 4a). The expression levels of mdm2 and PCNA were significantly lower at 3.2 µM when compared to 1.5 µM of OMH treatment after 48 h (Figure 4b). 

### 2.5. Effect of OMH on Protein Expression Levels

**Figure 5:**
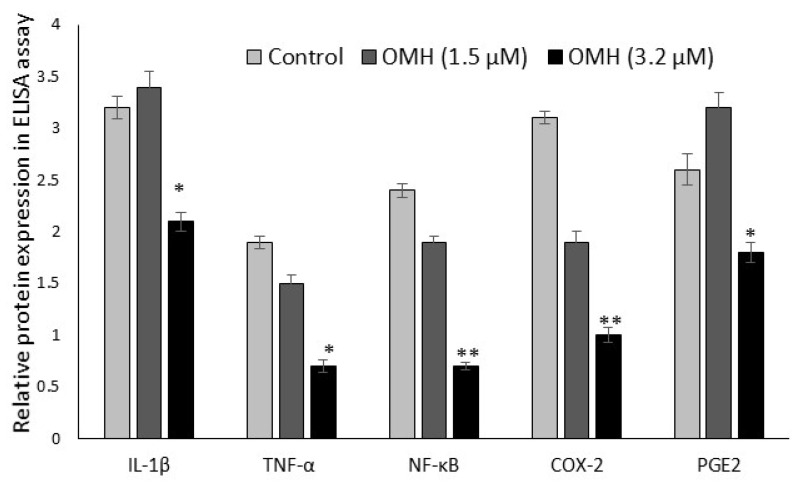
Effect of OMH on protein expression levels of IL-1β, TNF-α, NF-κB, COX-2, and PGE-2 in HT-115 cells after 48 h. Data presented as the means ± SD (*n* = 6). Values sharing a common superscript as * *p* ≤ 0.01 and ** *p* ≤ 0.05 compared with untreated HT-115 cells.

The protein expression levels of protumorigenic inflammatory cytokine (IL-1β, TNF-α, NF-κB, COX-2, and PGE-2) levels in the HT-115 cells after 48 h of OMH treatment are presented in Figure 5. OMH at a 3.2 µM dose significantly (*p* ≤ 0.01) decreased the cytokine levels of bowel-specific protumorigenic proteins COX-2 (two-fold) and PGE-2, TNF-α (1 fold) levels after 48 h. The protein levels of COX-2, PGE-2, and TNF-α were significantly (*p* ≤ 0.01) decreased in the OMH-treated cells when compared to the control. The inflammatory cytokine IL-1β and NF-κB levels also significantly decreased at the 3.2 µM dose of OMH-treated cells when compared to 1.5 µM of OMH and the control.

## 3. Discussion

Bioactive compounds from plant origins have been revealed to arrest the early and late stages of cancer progression, hence inhibiting the proliferation of malignant cancer cells [22]. Identifying potential anticancer bioactive compounds using in vitro bioassay systems is an essential way to identify new pharmaceutical research. We identified a phyto-active principle ononitol monohydrate (OMH), a glycosidic flavanol found with potential cytotoxic activity in HT-115 colorectal carcinoma cells. The tested higher dose (16 µM) of OMH potentially inhibited 84% of HT-115 cell growth. Further, the IC_50_ concentration of OMH was identified with 1.5 µM in 24 h and 3.2 µM in 48 h. In this context, many other flavone glycosides have been identified, such as kaempferol, morin, quercetin, 7-OH flavone, 5-OH flavone, 3-OH flavone, and flavone, with an apoptosis activity in colorectal cancer cell models and animal models [23,24].

The protein and gene expression levels of protumorigenic cytokine COX-2 were higher in the majority of colorectal carcinomas [25,26] and adenomas in animals and humans [13,27,28]. In our study, we observed an increased expression of COX-2 and PGE-2 in untreated HT-115 cells. The treatment with OMH significantly suppressed the expression of COX-2 and PGE-2 levels after 48 h, which confirmed the cellular uptake of OMH in the HT-115 cells, which downregulated the COX-2 levels. These low COX-2 levels effectively downregulate its major end product, PGE-2 synthesis from arachidonic acid. We confirmed that this might be an intracellular uptake of OMH, and its metabolites are directly proportional to decreased COX-2 levels, further lowering PGE-2 levels by inhibiting arachidonic acid utilization. The inhibition of COX-2 by pharmacological or genetic disruption results in a considerable decline in the size and number of adenomas in murine models of colonic carcinogenesis [29].

The mRNA expression levels of the OMH-treated HT-115 cells indicated that the cytosolic CYP1A levels were two-fold upregulated. In addition, OMH triggers the mitochondrial release of cytochrome c, which stabilize the fundamental oxido-reduction cycle in mitochondria [30]. The activation of CYP1A effectively controls the pro-oxidants and oxidative stress in colon cancer cells further, suppressing the proinflammatory cytokines IL-1β and TNF-α, which favors the deactivation of malignant cell apoptosis inhibitor NF-kB in colon cancer cells [26]. The observed antioxidant capacity neutralizes proinflammatory TNF-α/IL-1β, inhibiting protumorigenic COX-2/PGE-2 and stimulating the apoptosis mechanism via the inhibition of NF-kB, an apoptosis inhibitor [31,32]. OMH effectively maintains the balance between Bcl-2 and Bax (Bcl-2-associated X pro-apoptotic gene) and inclines the cells to apoptotic stimulation [33]. Further, the susceptibility of the OMH-treated cells to apoptosis tends to decrease TNF-α/NF-Kb expression, and increased levels of Bax/decreased levels of Bcl-2 due to the regulation of COX-2/PGE-2 [34].

Under normal conditions, mdm2 amalgamates the tumor-suppressor p53. Upon carcinogenesis, p53-mdm2 amalgamation is released by a tumor-suppressor gene, Cdkn2A [35]. Then, Cdkn2A suppresses the mdm-2 activity, leading to the release of p53, followed by the stabilization and accumulation of p53 in colon cancer cells [36]. Further, p53 controls the fate of cancer cell, which allow the cells to repair DNA damage or stimulate programmed cell death (apoptosis); it also regulates multiple genes, including caspases and p21, which are associated with the growth of cellular components [37]. The expression of caspase-3 has been considered to be an important terminal-cutting enzyme in apoptotic processes; it is regulated by multiple genes associated with pro-apoptosis and mitochondria-mediated apoptosis pathways [38]. We observed that the treatment with OMH significantly upregulated Bax, p53, and caspase-3, while Bcl-2 and mdm-2 were downregulated. Finally, the mechanism was apparent that OMH potentially suppresses proinflammatory TNF-α/IL-1β and protumorigenic COX-2, PGE-2 levels, which favors the activation and stabilization of p53, Bax expression via the inhibition of the apoptosis inhibitor NF-kB, which resulted in mitochondrial-mediated apoptosis. 

## 4. Materials and Method

### 4.1. Cell Lines and Molecular Biology Chemicals 

The HT-115 (human colon carcinoma cells) was obtained from American Type Culture Collections (ATCC), Manassas, Virginia, USA. Vero (kidney epithelial cells) and V79 (hamster lung fibroblast cell) cell lines were purchased from the National Center for Cell Sciences (NCCS), Pune, India. RPMI-1640 growth media, fetal bovine serum (FBS), penicillin and penicillin/streptomycin, and cell-culture-associated chemicals were purchased from Gibco, Paisley, UK. The cDNA synthesis kit was purchased from Qiagen, Hilden, Germany. SYBR Green PCR Master Mix was purchased from Qiagen, CA, USA. Propidium iodide, acridine orange, ethidium bromide, dimethyl sulfoxide, and trypsin were obtained from Sigma-Aldrich chemical company (St. Louis, MO, USA).

### 4.2. Cell Culture

The experimental cells, such as the HT-115, Vero, and V79 cells, were maintained in a 5% CO_2_ incubator at 37 °C and cultured using RPMI-1640 medium containing 10% FBS, 100 U/mL of penicillin, and 100 μg/mL of penicillin/streptomycin. According to the experimental requirements, the cell numbers and multiwell plates were selected and utilized for drug treatment with the respective period of incubation.

### 4.3. Extraction of Cassia Tora (L.) and Isolation of Ononitol Monohydrate

Ononitol monohydrate (OMH) was originally isolated from *Cassia tora* (L.) leaves upon sequential non-polar and polar solvent extraction and column chromatography methods [17]. Briefly, *Cassia tora* (L.) leaves were collected, shade-dried at room temperature, powdered, and subjected to serial extraction (1:3 ratio) with hexane, ethyl acetate, and methanol. The ethyl acetate extract was subjected to column chromatography over silica gel, then eluted initially with 100% hexane, hexane: ethyl acetate (range from 95:5 to 0:100), and finally with 100% ethyl acetate, and the fractions were collected. Based on the TLC patterns, the fractions were pooled together, and finally, 7 fractions were obtained. Fraction 4 formed as a crystal, and the crystal was subjected to X-ray crystallography and it was identified as ononitol monohydrate (The yield of OMH was 0.24%) [17]. In the present study, ononitol monohydrate (OMH) was kindly gifted from Dr. Ignacimuthu S.J., Director, St. Xavier’s Research Institute, Tamil Nadu, India.

### 4.4. Cytotoxicity Assay

To determine the cytotoxicity, the HT-115, Vero, and V79 cells were treated with increasing concentrations of OMH (0, 0.5, 1, 2, 4, 8, or 16 μM) in 96-well cell titer plates (Gibco, Grand Island, NY, USA) for 24 h and 48 h, respectively. The cytotoxic levels of OMH were determined using MTT (3-(4, 5-Dimethylthiazol-2-yl)-2,5-Diphenyltetrazolium Bromide) (Promega, Madison, WI, USA) [39]. A reference drug, fluorouracil, was tested with the same concentration to determine the comparative inhibitory effect between OMH and fluorouracil. After incubation, 20 μL of the MTT solution was added to each well in the dark and incubated for 4 h at 37 °C. Further, the viable cells producing purple formazan crystals were dissolved using DMSO, and the absorbance was measured at 492 nm using a microplate reader.

### 4.5. Cell and Nuclear Morphology

The morphological changes of the apoptotic characteristics of the OMH-treated HT-115 cells were observed and quantified using propidium iodide (PI) or acridine orange/ethidium bromide (AO/ErBr) fluorescent staining (Sigma-Aldrich, St. Louis, MO, USA) [40]. Briefly, the HT-115 cells (50,000) were seeded and cultured in a 24-well plate and treated with OMH (0, 0.75, 1.5 or 3.2 μM) for 48 h. Further, the OMH-treated cells were fixed in the same plate using 4% paraformaldehyde and stained with 1 mg/mL of PI or AO/ErBr at 37 °C for 15 min in the dark. Randomly, 300 stained cells were analyzed using an inverted fluorescence microscope (40× magnification), and the cells showing pathological changes were calculated manually. 

### 4.6. Quantitative Polymerase Chain Reaction (qPCR) Analysis

The cDNA was directly synthesized from the experimental cells’ total RNA using the Fastlane^®^ Cell cDNA kit (QIAGEN, Hilden, Germany) after 48 h, from the 1.5 and 3.2 μM dose of OMH-treated HT-115 cells, respectively. The transcription levels of protumorigenic inflammatory genes (IL-1β, TNF-α, NF-κB, COX-2, PGE-2), antioxidant- and tumor-suppressor-related genes (CYP1A, GSK3β, Cdkn2A, pRb1, p53, and mdm2), and apoptotic genes (Bcl-2, Bax, Caspase 3, CDKN1A, and PCNA) were quantified (Applied Biosystems, 7500 Fast, Waltham, MA, USA) using the QIAGEN real-time SYBR Green/ROX assay kit according to the kit protocol. Beta-actin was used as a reference gene. 2^−ΔΔCt^ methods were used to determine the relative mRNA expression level of a specific gene. Such as where ΔΔCt = (Ct, target gene of experimental group – Ct, β-actin of experimental group) − (Ct, target gene of control group − Ct, β-actin of control group) [41].

### 4.7. Inflammatory Cytokine Assay Using ELISA Method

Using the ELISA method, the quantities of cellular pro-inflammatory proteins, such as TNF-α, Nf-κB, COX-2, and PGE-2, were quantified from the OMH-treated (1.5 and 3.2 μM) HT-115 cells. Briefly, the HT-115 cells (50,000) were plated in a 24-well plate and treated with 1.5 and 3.2 μM of OMH at 37 °C. After 48 h incubation, the total cellular protein was extracted, and the levels of the TNF-α, NF-κB, COX-2, and PGE-2 protein levels were quantified according to the kit protocol (Qiagen, Hilden, Germany). The values were expressed as pg/mL of cells for TNF-α and NF-κB; ng/mL of cells for COX-2 and PGE-2.

### 4.8. Statistical Analysis

SPSS software/version-26 was used for the statistical significance analysis. The data are presented as mean values ± standard deviation (SD) and the significance between the treated groups (0.75, 1.5, or 3.2 µM doses of OMH) and the control groups (untreated). All of the results were four replicates in each group, and the differences are considered statistically significant at *p* ≤ 0.01 and *p* ≤ 0.05. The values were analyzed using a one-way analysis of variance (ANOVA) followed by Tukey’s test [42].

## 5. Conclusions

Ononitol monohydrate (OMH) potentially arrests HT-115 colon cancer cell growth. The inhibitory mechanism was associated with the cellular uptake of OMH, immediately arresting COX-2 expression and inhibiting secondary metabolite PGE-2 after controlling the pro-inflammatory and protumorigenic factors. OMH tends to trigger apoptosis by upregulating the expression of CDKN2A and Bax and activating p53 via nullifying mdm-2 and NF-kB. Taken together, the present findings demonstrate that OMH enhanced the apoptosis of HT-115 colon cancer cells; thus, it may be useful as an agent for the prevention and treatment of colon cancer. However, further detailed studies in animal models are required to explore the molecular mechanism’s in vivo response.

## Data Availability

Not applicable.
